# Are people in residential care entitled to receive rehabilitation services following hip fracture? Views of the public from a citizens’ jury

**DOI:** 10.1186/s12877-020-01575-y

**Published:** 2020-05-12

**Authors:** Maria Crotty, Emmanuel S. Gnanamanickam, Ian Cameron, Meera Agar, Julie Ratcliffe, Kate Laver

**Affiliations:** 1 Rehabilitation, Aged and Extended Care, College of Medicine and Public Health, Flinders University, GPO Box 2100, Adelaide, South Australia 5001 Australia; 2 NHMRC Cognitive Decline Partnership Centre, University of Sydney, Sydney, Australia; 3 Health Economics and Social Policy Group, Australian Centre for Precision Health, School of Health Sciences, University of South Australia, Adelaide, Australia; 4 John Walsh Centre for Rehabilitation Research, Faculty of Medicine and Health, University of Sydney, Sydney, Australia; 5 IMPACCT (Improving Palliative, Aged and Chronic Care through Clinical research and Translation) Centre, Faculty of Health, University of Technology Sydney, Ultimo, Australia; 6 Health Economics and Matthew Flinders Fellow, College of Nursing and Health Sciences, Flinders University, Adelaide, Australia

**Keywords:** Nursing homes, Dementia, Rehabilitation, Hip fracture, Citizens’ jury

## Abstract

**Background:**

Access to rehabilitation services for people living in residential care facilities is frequently limited. A randomised trial of a hospital outreach hip fracture rehabilitation program in residential care facilities has demonstrated improvements in mobility at four weeks and quality of life at 12 months but was not considered cost-effective by standard health economic metrics. The current study aimed to explore the general public’s views on issues involved in the allocation of rehabilitation resources for residents of care facilities.

**Methods:**

A citizens’ jury comprising 13 purposively sampled members of the general public, representative of the South Australian age, gender and household income profile. The jury considered the questions “Should there be an investment of physical rehabilitation services in residential care for older people following a hip fracture? If so, what is the best way of providing this service (considering funding, models of service delivery and equity)?” Deliberations were in the context of a state-wide health reform program. The jury was conducted over two days with an experienced independent facilitator, addressing questions developed by a steering group of research academics and clinicians.

**Results:**

The mean age of the citizens’ jury members was 43 (range 26 to 61). Eleven members voted for investment in outreach hospital rehabilitation services in residential aged care. All jurors agreed a number of strategies in addition to investment should be implemented, including health care planning and decision making, increased emphasis on hip fracture prevention, training of aged care staff in rehabilitation and routine provision of hospital discharge summaries to families. The jury further advocated for an increased focus on rehabilitation in residential care, potentially through accreditation criteria, increasing health literacy of residents and families, implementation of age friendly environment strategies and improving connections of care facilities with community, hospital and tertiary services.

**Conclusions:**

This citizens’ jury representative of the general public recommends that regardless of dementia and frailty, people who live in residential care and are walking and fracture their hips should have access to hospital outreach rehabilitation and recovery services.

## Background

Hip fractures are a common cause of loss of independence in older people and access to rehabilitation is inconsistent [1, 2]. National guidelines on the management of people with hip fracture suggest that those who live in residential care facilities should not be excluded from accessing rehabilitation services but in practice there is inconsistent access even for those who were walking prior to hip fracture [3, 4]. Hospital and community rehabilitation services who often operate in an environment of fiscal constraint, may not allocate rehabilitation services to people living in residential care facilities where the benefits are uncertain.

People who live in residential care facilities and fracture their hip often have comorbidities of dementia [5] and frailty [6]. They suffer particularly poor outcomes with high mortality rates and dramatic decreases in mobility [7, 8]. A retrospective study examining US Medicare data found that amongst people living in residential care facilities who were independently mobile pre-fracture, only 21% survived and regained their pre-fracture independence at a median of 4 months [9]. It is unclear whether these poor outcomes are the expected consequence of a significant morbid event occurring in a frail group or whether they resulted from a failure to access adequate recovery treatments.

The effectiveness of hip fracture rehabilitation for people living in residential care facilities is uncertain, in part because people from residential aged care facilities are not included in trials [10]. Similarly, evidence for people with dementia is scant. Multi-disciplinary rehabilitation services can improve outcomes for older people after hip fracture [11] and a co-ordinated program has been recommended [12]. Effectiveness has also been observed among those individuals with dementia [13] and providing organised rehabilitation after a hip fracture appears to increase the likelihood that an individual will recover independence [14]. Unfortunately, despite 40% of people with hip fractures also having cognitive impairment, very few trials (*n* = 7) have included people with dementia in examinations of the effects of rehabilitation after hip fracture [15]. One of the few randomised controlled trials examining hip fracture rehabilitation in aged care residents was completed in South Australia [16]. The trial recruited 240 residents of aged care facilities who were walking prior to hip fracture, almost all of whom were living with dementia. The trial examined the impact of a hospital outreach rehabilitation program. While gains in mobility were achieved at 4 weeks they were not sustained and despite small gains in quality of life sustaining at 12 months the program could not be considered cost effective using conventional health economic modelling [16].

As in many jurisdictions around the world, South Australia engaged in a health reform process in 2015–2016 [17] which involved reducing hospital infrastructure (including inpatient rehabilitation hospital beds), implementing out-of-hospital models of care (e.g. home rehabilitation and tele-rehabilitation) with the objective of achieving shorter inpatient stays and reviewing access criteria for services. During rapid health reform processes there is significant risk that the voices and preferences of people living in residential aged care, their carers and the general public are not heard. This led on to the citizens’ jury which was convened to explore issues involved in the allocation of rehabilitation resources for residents of care facilities. The aforementioned local trial results including information on cost effectiveness were available for discussion at the jury.

Citizens’ juries are a method for engaging members of the public in health policy decision making. Citizens (acting as jurors) deliberate pre-determined research questions and in doing so provide the perspectives of the broader public. Within health, citizens’ juries have been convened to address ethical issues, priority setting, policy, environmental health and wellbeing and have been conducted predominantly in Canada, USA, UK, Australia and New Zealand [18]. The purpose of recommendations stemming from the jury is to help policy makers understand the sentiments of the public and juries are considered particularly useful for addressing value-laden and controversial issues facing governments. Policy makers and health system planners in Australia have supported the use of citizens’ juries and they have been used to inform priorities and make recommendations for a number of health interventions including adolescent vaccination programs [19], surgical management of obesity [20], emergency department treatment [21] and taxing of soft drinks [22]. With respect to care for older people, a citizens’ jury conducted in the UK examined priorities for development of dementia care services [23] and another examined introduction of consumer directed care in residential care in Australia [24].

## Methods

This paper discusses the process and results of the citizens’ jury that addressed questions on providing rehabilitation for people living in residential care who fractured their hip. The jury were asked to consider the benefits and costs of rehabilitation in the context of the widely publicised state-wide health reform program [17] and constrained resources. The questions posed to the jury were: Should there be an investment of physical rehabilitation services in residential care for older people following a hip fracture? If so, what is the best way of providing this service (considering funding, models of service delivery and equity)? This study was approved by the Social and Behavioural Research Ethics Committee at Flinders University (project # 7141).

### Overview

**Fig. 1:**
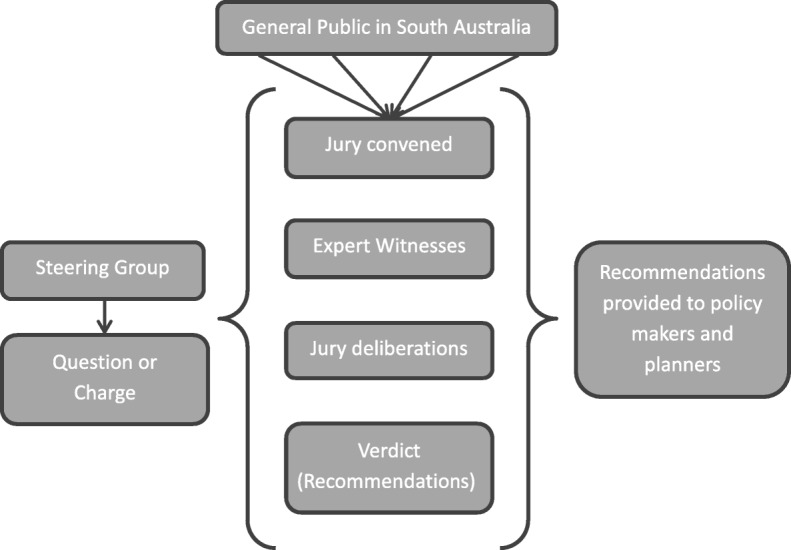
Overview of jury process

An overview of the jury process is shown in Fig. 1. A steering group consisting of research academics and clinicians was formed and was responsible for developing and refining the question (‘charge’) for the jury and developing the program of speakers and topics. The jury took place over 2 days (Saturday/Sunday) in 2016. A purposively sampled group of members of the public were appointed to the jury following informed consent to participate and were presented with the question. On the first day the jury had the opportunity to listen to a variety of expert witnesses who provided the jury with background information to assist their decision making and presented their views on the problem. The second day was dedicated to deliberation and the formulation of recommendations. The two-day jury process was facilitated by an experienced independent facilitator.

### Participants (‘jurors’)

Participants were recruited from the general public in South Australia through a market research company. The criteria for selection of jury members were that they should be representative of the general population in South Australia in terms of age, gender and household income. Those currently working in the field of health and aged care were excluded and so were those who were a primary carer for someone with dementia. The market research company used a list of verified landline and mobile phone numbers to source participants. Eligibility of the participants, relative to the criteria, was determined by short telephone survey.

### Speakers (‘expert witnesses’)

Expert witnesses identified by the steering group included medical experts on palliative care, rehabilitation and hip fracture, families of people who lived in residential care and had suffered a hip fracture describing their experience, providers from residential care who explained staffing and funding issues, an expert on ethical decision making and a health economist discussing issues relating to efficiency and equity in the allocation of scarce health care resources [25, 26]. The local trial results were presented demonstrating limited cost effectiveness of a hospital outreach multi-disciplinary rehabilitation intervention delivered in residential care for residents recovering from a hip fracture. The tensions between end of life care and maintaining function and quality of life in residential aged care facilities were discussed. Jurors were able to interact with the witnesses in dedicated question times and then discuss the issues as a group. The jurors considered the issues in the context of end of life care and quality of life in people with dementia and the health economist and palliative care expert provided advice regarding this. The jury then deliberated and delivered their verdict (answers to the questions) [27].

### Data collection and analysis

The information provided by the witnesses, the questions raised by the jury members and the intermediary and final presentation of recommendations of the jury together formed the data for this paper. Audio recordings of the formal discussions of the jury were collected. The final recommendations of the jury were presented by one of the jury members and video recorded. A thematic analysis of both the intermediary and final recommendations of the jury was conducted to draw out the themes that underpinned the reasoning behind the jury’s recommendations [28]. Jurors were given the opportunity to evaluate the entire process for satisfaction, amount of information, quality of witnesses and bias in information and guidance provided.

## Results

### Participants

Fourteen members of the public were recruited to the jury although one member withdrew before the weekend of the jury. The final jury consisted of 13 participants with a mean age of 43 years and ranging from 26 to 61 years of age (Table 1).

**Table 1:** Characteristics of Jury members

Characteristic		N (%)
Mean age (SD)	43 (12)	
18–34 year olds		5 (38)
35–54 year olds		4 (31)
55–70 year olds		4 (31)
**Gender**		
Male		7 (54)
Female		6 (46)
**Paid Work**		
Yes		10 (77)
No		3 (23)
**Annual Income**		
< $50,000		5 (38)
$50,000 – $100,000		4 (31)
> $100,000		4 (31)
**Highest educational qualification attained**		
Primary School		1 (8)
High school		4 (29)
Technical College, or trade certificate or diploma		6 (32)
University or other tertiary institute degree		2 (14)
**Born in Australia**		
Yes		11 (85)
No		2 (15)

### Jury recommendations

The jury recommendations are presented in Table 2 and Table 3. For the question on investment in physical rehabilitation services in residential aged care following a hip fracture (Table 2), 11 jury members voted that there should be an investment while the two remaining members voted no. Both those voting ‘no’ and those voting ‘yes’ identified similar issues that needed to be considered by policy makers (elaborated in the next section).

**Table 2:** Question 1 - Should there be an investment of physical rehabilitation services in residential care for older people following hip fracture?

Verdict votes	Buts (common to both the yes and no groups)
0 voted yes 0 voted no 11 voted ‘yes, but…’ 2 voted ‘no, but….’	• There is need to invest in other options such as good advance health care planning, family education, ethical decision making and that surgery may not be the best option • There is need to invest in prevention to reduce falls/injuries • Transparent eligibility criteria are essential – not everyone should receive rehabilitation, for example people not walking prior to hip fracture • Training for residential care staff on how to promote independence • An opt out option of treatments should be available to everyone • Funding for residential care should reward functional gain, not dependency • The rehabilitation program provided should be flexible. For example, a strong psycho-social focus may be needed rather than a physical focus • Provide discharge summary to family member prior to return to residential care to assist with transition

**Table 3:** Question 2 –what are the best models

Characteristics of the rehabilitation service	Recommendation	Voting
Location	Best venue for rehabilitation was in the residential care facility	All agreed
Type	The investment should be in the form of an in-reach service	12 jurors agreed. 1 juror had reservations.
Attributes	Flexibility, regular review, tailored to individual, capped in length, multidisciplinary, holistic, respectful and equitable	All agreed
Decision on content	Option 1: Decision making should be shared between a rehabilitation ‘expert or broker’ and the resident and/or family to ensure informed choice. Option 2: There should be a menu of a few set options which families could select from e.g. physical therapy, geriatrician, dietician	11 jurors preferred option 1 and 2 jurors preferred option 2
Workforce investment	There should be an investment in up skilling residential care workers in rehabilitation principles and practice	All agreed
Communication and information	Better communication between the discharging hospital and the residential care facility	All agreed
Funding	Option 1: Government funding shared between Australian and State governments Option 2: State government Option 3: Australian government	9 jurors preferred option 1, 2 jurors option 2, one juror option 3 and 1 juror said that they did not know

All jurors agreed that investment in rehabilitation alone was not the answer and that a number of strategies should be implemented (‘buts’). This included the need for appropriate health care planning and decision making, through the use of advance care planning and educating and supporting families to make early decisions, starting prior to the surgery, on post fracture care including whether surgery would lead to the best outcome for the person. The jury recognised the high incidence of hip fracture in residential care and the burden associated with this and thus recommended an increased emphasis on hip fracture prevention. The jury also felt that staff within aged care facilities should be provided with more training in rehabilitation skills; this would complement the work provided by specialist rehabilitation services and be beneficial for resident wellbeing more broadly. The jury emphasised the importance of good communication (between the hospital and both the residential care facility and family members) and suggested that hospitals routinely provide the discharge summary and information on optimal post hip fracture care to a family member in addition to aged care facility staff; this is not current practice in Australian hospitals where the discharge summary is provided to aged care facility staff. The reasoning behind this was that if family members had more information about optimal care, they may be able to play a role in the rehabilitation of the person by encouraging mobility and be better placed to advocate for required services.

Table 3 presents a summary of the jury’s recommendation on the best models to provide the rehabilitation services. Included are the jury’s recommendations on various themes of the rehabilitation service such as location, type, decision making, funding and other issues.

In addition to the recommendations on the two questions that were posed to the jury, the jury identified additional strategies that would complement the recommendations. The jury advocated for:

1. An increase in rehabilitation focus in residential care for people returning from hospital potentially through accreditation criteria supporting this approach.
2. Increasing health literacy of residents/families in order to improve understanding of hip fracture, rehabilitation services, advance care planning and decision making.
3. Implementing strategies that enable age friendly environments, encouraging people to keep active and remain socially connected
4. Improving connections to the local community including a schools and community groups, hospitals with geriatricians and specialist therapists.

### Jury reasoning

The data from the jury’s deliberations were analysed to evaluate the values and reasoning that underpinned their recommendations. Equity was an important value of the deliberations. For the question on investment in rehabilitation service after a hip fracture, jury members felt that people who had worked hard and contributed to society all their lives should not be denied access to rehabilitation in part because they were residing in a nursing home. Conversely, those (*n* = 2) who opposed the investment expressed concerns about the relative cost ineffectiveness of the programme in comparison with other potential investments for scarce dollars and felt that the program should not focus on physical rehabilitation alone, but holistic rehabilitation instead.

Three themes were important in the reasoning: person centred care, quality of care and economic considerations. These three themes were overlapped in some deliberations and underpinned each other in other deliberations.

Person centred care underpinned several aspects or characteristics of the rehabilitation service detailed in Table 3. Values such as familiarity, comfort and dignity underpinned the recommendation that the care home is the best location for the service. Patient centric values also underpinned shared decision making for service provision and highlighted communication and information sharing as a key component of the service. This theme also underpinned quality of care, one of the other two overarching themes from the data. Several ethical and quality of care aspects were commonly discussed by jurors. These include equipping of carers, communication and information, shared decision making and care planning that is patient centric, additional approaches to complement the service such as falls prevention, access to a rehabilitation mediator, multi-disciplinary holistic care and regular patient centric review of care provided.

The economic considerations that formed the basis for the recommendations included optimal use and allocation of resource use, the government’s responsibility for the provision and funding of care for this vulnerable population group and the need to include a programme of recovery after surgery.

Data from the evaluation surveys for jurors showed that 69% were very satisfied and 31% were satisfied with the overall process. All participants were either satisfied or very satisfied with the background information provided, the expert witness presentations and the deliberations.

## Discussion

Demographic changes associated with aging are associated with an increasing demand for long term residential care and maintaining quality of life in this setting is likely to involve access to rehabilitation to preserve function particularly after illness. The world report on ageing and health [29] calls for action in enabling older people to access rehabilitation services. Against a backdrop of high and growing demand for rehabilitation services, there is uncertainty about “the return on investment” of providing these services to people living in residential care nearing the end of life. Despite being presented with information on the low “return on investment” by a health economist, the majority of jurors favoured the provision of rehabilitation services to people who were old, at the end of life and frequently diagnosed with dementia, based on the principles of equity and maintaining quality of life. The citizens’ jury recommendations emphasised choice, access and family involvement. Concerned that without rehabilitation, a frail older person would find it difficult to recover their ability to walk the jury members generally favoured clear criteria and judicious allocation of programs to those who were likely to recover walking and only where appropriate the provision of palliation.

The findings confirm other work showing that recovery and rehabilitation services are highly valued by members of the public and consumers of health services. Great importance is placed on maintaining the ability to walk and many health service consumers believe that maintaining walking is strongly associated with quality of life [30]. Even in the very old, a discrete choice experiment involving families and patients with recent experience of hip fractures found that all participants including those from residential care disagreed with the statement ‘I would prefer to go into a wheelchair now and forget about walking’ and they were prepared to suffer moderate discomfort to recover walking [31].

The proportion of people living in residential care facilities prior to death varies across countries. About one fifth of older people (65+) in England and Wales [32] live in residential aged care prior to death while in Australia this proportion is approximately one third of all deaths among older people. There have been calls to treat hip fracture as a trigger for an “end of life” model of care [33] and in Australia residential aged care plays an important role in end of life care. Whilst acknowledging the importance of advance directives this jury felt that care which promoted recovery from hip fracture in a residential care setting was appropriate.

These findings have implications for policy makers designing funding models for people living in residential care. Treating people with hip fractures requires shared decision making; planning and coordination of surgical, anaesthetic, medical, rehabilitation, nursing and social work services across a range of care settings some of which are funded by the Australian national government and the others by the state governments. People who live in residential care access services in a different way to those living in the general community and they currently access substantially less rehabilitation services. It could be argued that once a decision has been made “to fix the hip” by operating, the older person should be offered various levels of support with recovery (including rehabilitation) to ensure an effective treatment outcome is achieved. While the cost of surgical treatment of a hip fracture can be between AU$20,000 and AU$34,000, without some post-operative investment in physiotherapy and nutrition services to promote recovery, a patient may be at increased risk of frailty, falls and re-fracture [34].

Research evaluating health services for older people receives relatively little funding relative to other health conditions [35]. Although our jurors were relatively young (ranging from 26 to 61 years) they showed a high level of support for investment in care of older people in residential care settings. Application of citizens' jury methodology to assist with prioritisation of research funding (within a context of scarce resources) would be a useful approach for funding bodies to understand research priorities of members of the public and assist with making decisions about allocation of funds.

The analysis of the recommendations from the citizens’ jury in conjunction with a qualitative analysis of the reasoning underpinning the recommendations reveals the value of a citizens’ jury process. It provides critical information to support the future involvement of members of the general public in such processes to informing policy and decision making. The jury members were able to formulate informed recommendations based on the detailed and complex information provided to them through the witnesses (experts). While some of the recommendations such as patient centred care are already part of guidelines e.g. in Scotland [36] jury members called for stronger connections between aged care, community and hospitals suggesting that intersectoral integration is a high priority.

## Conclusions

This citizens’ jury recommends that regardless of dementia and frailty, people who live in residential long-term care and are walking and fracture their hips should have access to hospital outreach rehabilitation and recovery services. Whilst these reasoned views mirror to some extent existing recommendations and guidelines on the provision of rehabilitation care in residential care (e.g. NICE Hip Fracture Guidelines), they also confirm that a representative group of the general public supports investment in mobility and quality of life even when people are living in institutions.

## Data Availability

All data generated or analysed during this study are included in this published article [and its supplementary information files].
